# Identifying important nodes in complex networks based on extended degree and E-shell hierarchy decomposition

**DOI:** 10.1038/s41598-023-30308-5

**Published:** 2023-02-23

**Authors:** Jun Liu, Jiming Zheng

**Affiliations:** 1 School of Science, Chongqing University of Posts and Telecommunications, Chongqing, 400065 China; 2 Key Lab of Intelligent Analysis and Decision on Complex System, Chongqing University of Posts and Telecommunications, Chongqing, 400065 China

**Keywords:** Complex networks, Nonlinear phenomena, Computational science

## Abstract

The identification of important nodes is a hot topic in complex networks. Many methods have been proposed in different fields for solving this problem. Most previous work emphasized the role of a single feature and, as a result, rarely made full use of multiple items. This paper proposes a new method that utilizes multiple characteristics of nodes for the evaluation of their importance. First, an extended degree is defined to improve the classical degree. And E-shell hierarchy decomposition is put forward for determining nodes’ position through the network’s hierarchical structure. Then, based on the combination of these two components, a hybrid characteristic centrality and its extended version are proposed for evaluating the importance of nodes. Extensive experiments are conducted in six real networks, and the susceptible–infected–recovered model and monotonicity criterion are introduced to test the performance of the new approach. The comparison results demonstrate that the proposed new approach exposes more competitive advantages in both accuracy and resolution compared to the other five approaches.

## Introduction

With the rapidly growing scale of networks, topics concerning complex networks are emerging in network science^1,2^. Generally, complex networks are modeled by various systems in the real world, which are disorganized, self-similar, and small-world^3^. Massive systems can be remolded by complex networks, for instance, protein networks in biology^4^, social networks in sociology^5^, financial networks in economics^6^, power networks in engineering^7^, and so on. Complex networks have received extensive attention in theory and practice in recent years^8–10^. The identification of important nodes has become a fundamental problem in complex networks^11–13^, which has not only theoretical value^14,15^, but likewise practical applications. Some examples can be easily found. In social networks, the positive contribution of “celebrities” can effectively reduce the spread of negative social opinion. Ad-technology vendors often seek out the most influential users for advertising to maximize revenue in marketing networks. More applications can be given in other cases, such as disease control^16^ and sociology^17^.

The identification of important nodes in complex networks is an NP-hard problem^18^. Up to now, many schemes with polynomial complexity have been presented. For example, degree centrality^19^ counts the number of node’s nearest neighbors and considers important nodes to have more neighbors. Closeness centrality^19^ considers the average length of the shortest path from the target node to other nodes in the network. Betweenness centrality^19^ calculates the fraction of the shortest paths that cover the target node. A number of variants and approximations have been proposed for speed and accuracy^20–22^. These schemes, which are called “classical”, exploit the topological characteristics of nodes while ignoring community properties. However, it is well known that community organization is a main feature of complex networks^23^.

Consequently, when one considers community organizations, nodes that are not of interest in classical centrality may have potential influence. Community centrality considers the heterogeneity between intra-community links and inter-community links^24–26^. The intra-community links quantify the local influence of a node inside a community. Conversely, inter-community links account for the global influence of nodes on various communities. Many ideas of community centrality have been put out in light of the various ways in which the two type of links might be coupled^27–29^. Zhao et al.^27^ distinguish the weights of inter-community links by community size. The community-based mediator targets influential nodes through entropy and normalized degree^28^. This entropy is generated by the heterogeneity of the links between the inside and outside communities. Modular Vitality assesses the centrality of a node by investigating the modular variations caused when one is removed^29^.

Besides the community, the hierarchical structure of the network has also attracted attention^30^. Some previous outcomes have used the hierarchical structure to drive the K-shell decomposition algorithm and its improvements^31–34^. Although the K-shell algorithm has promising applications in various fields^35–38^, there has been a drive to improve its resolution. This is because it ties many nodes to the same shell, even though these nodes differ in influence^39,40^. Zeng et al.^41^ introduced the exhausted degree and proposed a mixed degree decomposition (MDD) method to identify important nodes. Bae et al.^42^ presented an extended version, called neighborhood coreness, using the k-shell index of 1-order neighborhood nodes. Feng et al.^43^ consider opinion leaders and structural hole nodes to have more ability to influence others. Wang et al.^44^ suggested a integral k-shell algorithm (IKS) by accumulating historical k-shell index and second-order degree of neighborhood. Liu et al.^45^ refined the K-shell algorithm utilizing TOPSIS technique.

Many facts indicate that the importance of the node depends on multiple characteristics, such as degree, neighbors, position, and so on. Sheikhahmadi et al.^46^ presented a multi-criteria approach (MCDE) which utilizes a combination of node’s degree, k-shell index and information entropy. There is still relatively little work on multi-characteristic methods. As a result, a new approach based on multiple characteristics is proposed in this paper. The interests of our work are as follows,

1. An improved version of the classical degree, extended degree, is introduced.
2. A E-shell hierarchy decomposition is put forward for determining nodes’ position information through the network’s hierarchical structure.
3. A hybrid characteristic centrality (HCC) is presented, which combines the extended degree and E-shell hierarchy decomposition.

Extensive experiments were performed in six real networks and the performance of the proposed approach was examined using the monotonicity function^42^ and the susceptible–infected–recovered (SIR) model^47,48^. The results indicate that the new approach is more competitive than the classical and community centrality in terms of accuracy and resolution.

The framework of this paper is organized as follows. The “Preliminaries” section briefly introduces some basic preliminaries. The “Methods” section presents the new method and provides a simple example. The “Experiments” section examines the performance of the new method and compares the existing algorithms. The “Discussion” section summarizes the work.

## Preliminaries

Let $G=(V,E)$ be an unweighted undirected network, where *V* and *E* are the set of nodes and edges, respectively. Denote $n=|V|$ and $m=|E|$. The adjacency of network *G* can be represented by $A=(a_{uv})_{n\times n}$, where $a_{uv}$ indicates the connection between nodes *u* and *v*. $a_{uv}=1$, if nodes *u* and *v* are directly connected; $a_{uv}=0$, other cases.

### K-order neighborhood

For any two nodes *u* and *v*, *v* is said to be an *k*-order neighbor of *u* if there exists the smallest positive integer such that Eq. (1) holds,

1 $$\begin{aligned} \prod \limits _{i=0}^{k-1} a_{v_{i}v_{i+1}} =1 \end{aligned}$$

where $v_{i}\in G \ (i=0,\ 1,\,\ldots ,\ k)$, $v_{0} =u$ and $v_{k} =v$. Denote the k-order neighborhood of node *u* as the set $\phi ^{(k)} (u)$, which consists of all k-order neighbors of *u*. If not otherwise specified, note that $\phi ^{(1)}(u)=\phi (u)$. The number of 1-order neighbors of node *u* is called its degree^19^ and is denoted by *k*(*u*), i.e.,

2 $$\begin{aligned} k(u)=|\phi (u)|\end{aligned}$$

### K-shell hierarchy decomposition

The main idea of the K-shell hierarchy decomposition: for a given *k*, a subgroup of the network called *k*-shell is obtained by iteratively deleting nodes with degree less than or equal to *k*. All node within the *k*-shell have the same index *k*. If there are no isolated nodes (degree equal to 0) in the network, then the nodes with degree equal to 1 have the lowest importance. Therefore, these nodes and their connected edges are deleted from the current network. Again, the new nodes with degrees less than or equal to 1 and their connected edges need to be deleted. Continue the above process until the degree of each node is greater than 1 in the current network. The nodes deleted in this round form 1-shell. Based on the above description, the procedure of the K-shell algorithm can be summarized as follows:

*Step 1* delete nodes with degree *k*.

*Step 2* repeatedly remove the new nodes whose degree is less than or equal to *k* until the degree of each new node is greater than *k*.

*Step 3* all nodes deleted in steps 1 and 2 form the *k*-shell.

*Step 4* let $k=K=1$, and repeat the above steps. In the above program, if the initial and maximum values of *k* are 1 and *K* respectively, then we can get *K* shells, i.e., 1-shell, 2-shell, ..., *K*-shell.

### Susceptible–infected–recovered (SIR) model

The SIR model is a classical model of disease transmission in which individuals are classified into 3 states: susceptible (S), infected (I), and recovered (R). Initially, a single (or multiple) target individual is selected and its state is specified as I. At each step, the infected individuals spread the disease to each of its susceptible 1-order neighbors and enters state recovered. Herein, the infection rate and recovery rate are $\alpha$ and $\beta$, respectively. Then, the spread procedure is continued until there are no individuals with infected state in the network. The final cumulative count of infected individuals is considered as the real spread ability of the initial target individuals.

## Methods

### Main idea

Degree centrality and K-shell decomposition are two traditional single-characteristic schemes. However, numerous experiments have demonstrated that using a single characteristic to measure the importance of nodes is unreliable. Accordingly, a new idea is to combine multiple characteristics. Previous works have simply combined degree centrality and K-shell decomposition, but this fails to address the underlying problem: their low resolution limits the performance of the combination. Based on the above consideration, the interest of our work lies in the improvement of the classic degree and the K-shell decomposition, as well as their combination. As a result, we propose an extended degree and an E-shell hierarchy decomposition, and a combination of them called hybrid characteristic centrality.

### Extended degree and E-shell hierarchy decomposition

The classical degree only counts the number of neighbors of the node itself. Thus, we define the extended degree to overcome the limitation of classical degree. Let *G* be an unweighted undirected network. The degree and 1-order neighbors of node $u\in G$ are denoted as *k*(*u*) and $\phi (u)$, respectively. Then, the extended degree of node *u*, denoted by $k^{ex}(u)$, is defined by

3 $$\begin{aligned} k^{ex}(u)= \delta *k(u)+ (1-\delta )*\sum \limits _{v\in \phi (u)} k(v) \end{aligned}$$

where $\delta \in [0,1]$ is a weight which reflects the dependence of $k^{ex}(u)$ on *k*(*u*). If $\delta =1$, then Eq. (3) degenerates to the classical degree (Eq. 2).

Next, we propose a E-shell hierarchy decomposition to determine the position of each node by decomposing the network’s hierarchical structure. In this method, the nodes with minimum extended degree are found and deleted from the current network in each iteration. Then, these nodes are tagged with a position index, which is represented here by the iterations number. The procedures of E-shell hierarchy decomposition are described below.

*Step 1*:Input a network *G*.

*Step 2* Initialize the iteration number $p=1$ and $G_{1}=G$.

*Step 3* Find the set of minimum nodes $S_{p}=\mathop {\arg \max }\nolimits _{u\in G_{p}} \{k^{ex}(u)\}$.

*Step 4* Tag a position index $index_p$ for each node within $S_p$, where $index_p=p$.

*Step 5* Remove $S_p$ from $G_p$ and then get a new network $G_{p+1}$. If $G_{p+1}$ is non-empty, proceed to step 6; otherwise, the procedure terminates.

*Step 6* Update the extended degree of each node within $G_{p+1}$.

*Step 7* Update the iteration number $p=p+1$.

*Step 8* Return to step 3.

### Hybrid characteristic centrality and its extension

The hybrid characteristic centrality combines the extended degree and the E-shell decomposition. Because of this, it gives a better understanding of how important a node is. On the one hand, the extended degree measures the local influence using degree about the node itself and its neighbors. On the other hand, E-shell decomposition reflects the global influence of a node by its position in the network. In brief, a node receives greater importance by having more neighbors and a higher position. Let $k^{ex}(u)$ be the extended degree of node *u*. Denote the position index of node *u* given by E-shell decomposition as *pos*(*u*). The HCC of node *u*, written by *HCC*(*u*), can be calculated by

4 $$\begin{aligned} HCC(u)=\frac{k^{ex}(u)}{k_{max}^{ex}}+\frac{pos(u)}{pos_{max}} \end{aligned}$$

where $k_{max}^{ex}$ and $pos_{max}$ signify the maximum extended degree and position index of the nodes, respectively. Further, EHCC, an extensive version of HCC, is introduced as follows.

5 $$\begin{aligned} EHCC(u)=HCC(u)+\sum \limits _{v\in \phi (u)} HCC(v) \end{aligned}$$

EHCC draws on the idea of the extended degree, but it utilizes more information about a node, which includes the degree and position of its neighbors as well as itself. Nodes with higher EHCC values are commonly rewarded with a greater importance.

### Computational complexity

The complexity of the EHHC calculation is as follows.

1. The complexity of calculating the extended degree is *O*(*n*).
2. The complexity of the E-shell procedure for determining the position of the node is *O*(*m*) (similar to K-shell).
3. Therefore, the total complexity is $O(n+m)$.

### Computational process

**Figure 1:**
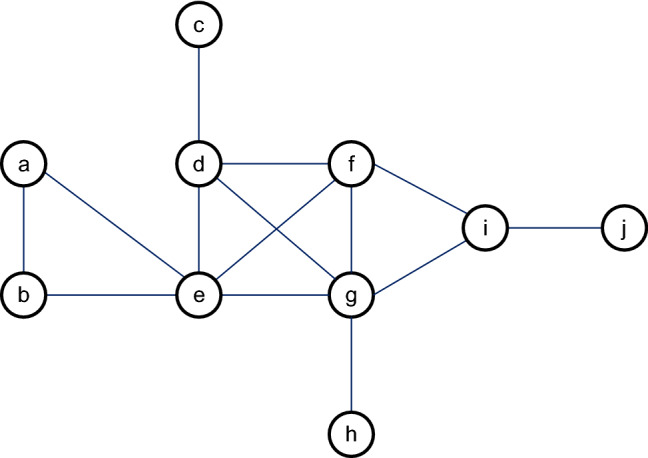
A simple network.

To interpret the calculation procedure of the new method, a simple example is given. Figure 1 shows a simple network with 10 nodes and 14 edges.

First, the extended degree of each node is calculate by Eqs. (2) and (3). Take $\delta =0.5$ in Eq. (3). The computational results are provided in Table 1. In the case of node *a*, the extended degree is $k^{ex}(a)=0.5*k(a)+0.5*(k(b)+k(e))=4.5$.

**Table 1:** The class degree and extended degree of each node in the simple network.

Node	*a*	*b*	*c*	*d*	*e*	*f*	*g*	*h*	*i*	*j*
Classical egree	2	2	1	4	5	4	5	1	3	1
Extended degree	4.5	4.5	2.5	9.5	11	10.5	11	3	6.5	2

Second, the position index of each node is obtained according to the E-shell decomposition procedure. The complete process listed in Table 2. We observe that the position index of node *a* is 4.

**Table 2:** The E-shell decomposition procedure of the simple network.

Iteration number	Minimum extended degree	Delete node set	Position index
1	2	$\{j\}$	1
2	2.5	$\{c\}$	2
3	3	$\{h\}$	3
4	4.5	$\{a,b\}$	4
5	5	$\{i\}$	5
6	6	$\{d,e,f,g\}$	6

Third, the HCC of each node is computed by Eq. (4). From Tables 1 and 2, the maximum extended degree and the maximum position index are 11 and 6, respectively. As a result, HCC of nodes *a* is $HCC(a)=\frac{4.5}{11}+\frac{4}{6}=1.08$.

At last, the EHCC value of each node is computed using Eq. (5). The computation of node *a* is $EHCC(a)=HCC(a)+HCC(b)+HCC(e)=4.15$. The results of HCC and EHCC are given by Table 3.

**Table 3:** The results of EHCC and HCC for the simple network.

Node	*a*	*b*	*c*	*d*	*e*	*f*	*g*	*h*	*i*	*j*
HCC	1.08	1.08	0.56	1.86	2	1.95	2	0.77	1.42	0.35
EHCC	4.15	4.15	2.42	8.38	9.97	9.24	10.01	2.77	5.73	1.77

## Experiments

### Experimental setup

Experimental environment: Windows 10 system with Intel(R) Core i7-12700H (2.1 GHz), 16 GB RAM and 512 GB Hard Disk. The methods and experiments were implemented in Python 3.11.0.

### Data sets

Six types of networks were selected for the experiment. A social network of dolphin populations (Dolphins)^49^. A metabolic network of C. elegans (Celegans)^50^. A social network for exchanging e-mails (Email)^51^, a power network in the western United States (Power)^52^. A collaborative network of Arxiv paper authors (GrQc)^53^. A relational network of PGP users (PGP)^54^. These data are available from the https://networkrepository.com/. The topological parameters of these networks are listed in Table 4.

**Table 4:** Topological parameters of six real networks: node number *n*, edge number *m*, maximum degree $k_{max}$, and spread threshold $\alpha _{th}$^55^.

Network	Type	*n*	*m*	$\langle k\rangle$	$k_{max}$	$\alpha _{th}$
Dolphins	Community	62	159	5.12	12	0.1470
Celegans	Metabolic	297	2148	14.46	134	0.0384
Email	Social	1133	5451	9.62	71	0.0535
Power	Power	4941	6594	2.67	19	0.2583
GrQc	Collaboration	5241	14,484	5.52	81	0.0593
PGP	Relationship	10,680	24,316	4.55	205	0.0530

### Comparison algorithm

The proposed method is compared with five well-known methods, as shown in Table 5.

**Table 5:** The proposed method and five well-known methods.

Method	Type	Complexity
Degree^19^	Local	*O*(*n*)
K-shell^22^	Global	*O*(*n*)
WKSDN^28^	Combined	$O(n+m)$
CHB^24^	Community	$O(n^2)$
MCDE^46^	Multiple	$O(n+m)$
EHCC (our method)	Multiple	$O(n+m)$

### Evaluation indicators

#### Accuracy

Accuracy is a criterion to evaluate the algorithm’s performance. Inspired by previous works^41,42,44,46^, the accuracy of the algorithm was investigated by the SIR model in this experiment. Here, we conducted 500 simulations for each node and used its average value as the final results. Let $\sigma _{u}$ and $x_{u}$ be the ranking of node *u* provided by the SIR model and algorithm *X*, respectively. The ranking sequences $\sigma$ and *x* are sorted in descending order. In order to quantify the accuracy of algorithm *X*, we take the sequence $\sigma$ as the benchmark and calculates the consistency coefficient between the ranking sequences $\sigma$ and *x*. For this purpose, a pair of nodes *u* and *v* is considered as follows:

- if $(x_{u}-x_{v})\times (\sigma _{u}-\sigma _{v})>0$, then nodes *u* and *v* are said to have a positive relationship in *x*.
- if $(x_{u}-x_{v})\times (\sigma _{u}-\sigma _{v})<0$, then nodes *u* and *v* are said to have a negative relationship in *x*.
- if $(x_{u}-x_{v})\times (\sigma _{u}-\sigma _{v})=0$, then nodes *u* and *v* are said to be independent in *x*. Based on the above, the consistency coefficient of ranking sequence *X*, denoted by *Coe*(*X*), is defined as
  6 $$\begin{aligned} Coe(x)=\frac{2\times (n_a-n_b)}{n\times (n-1)} \end{aligned}$$
  where *n* is the number of nodes, $n_a$ and $n_b$ represent the number of node pairs with positive and negative relationships in the ranking sequence *x*, respectively. Obviously, $Coe(x)\in [0,1]$. $Coe(X)=1$ means that each node pair has a positive relationship and thus the algorithm *X* has the best accuracy. The worst case is $Coe(X)=0$.

#### Resolution

There may be multiple nodes with the same ranking in the ranking sequence. For this reason, we introduce the cumulative distribution function (CDF) to describe the distribution of nodes. Let *x* be the ranking sequence generated by algorithm *X*. Let $\omega \in [0,1]$ be the identity of the nodes, then the mathematical equation of $CDF(\omega )$ can be written by

7 $$\begin{aligned} CDF(\omega )=\frac{n_{x,\omega }}{n} \end{aligned}$$

where $n_{x,\omega }$ signifies the number of nodes whose identity is greater than or equal to $\omega$ in sequence *x*. Here, the identity a node is the reciprocal of the number of nodes with the same ranking. Obviously, the faster the CDF increases, the greater the number of nodes with high identity, and the higher the resolution.

The monotonicity function measures the resolution by calculating the fraction of the number of nodes that rank differently^42^. Let $d_x$ be the count of nodes in *x* that are distinguishable (nodes with different rankings are distinguishable). The monotonicity of sequence *x* is calculated as follows

8 $$\begin{aligned} Monotonocity(x)=\left( 1-\frac{\sum \nolimits _{i=1}^{d_x} n_{i}\times (n_i -1)}{n\times (n-1)}\right) ^{2} \end{aligned}$$

where $n_i$ denotes the number of nodes ranked *i* in sequence *x*. $Monotonicity(x)=1$ indicates the best monotonicity of the ranking sequence *x*. $Monotonicity(x)=0$ means that the ranking of all nodes is the same and monotonicity is the worst in this case.

### Experimental results

#### Results of accuracy

Table 6 reports the consistency coefficients (Eq. 6) between the different algorithms and the SIR model in the six real networks. Here, the infection rate $\alpha =1.05\times \alpha _{th}$ and the recovery rate $\beta =1$ are chosen in the SIR model. The numerical results show that, except for the network GrQc, EHCC provides the most accurate results compared to the other five methods. In the network GrQc, MCDE performs slightly better than EHCC. It is also noted that EHCCC still outperforms the other four methods. In the networks Email, Power, and PGP, EHCC has the highest consistency coefficients, followed by MCDE. In networks Dolphins and Celegans, EHCC and WKSDN have the best and second best performance respectively. Moreover, we observe that the results of the K-shell method are the worst in all networks.

**Table 6:** The consistency coefficients between the node ranking sequences of the different algorithms and the ranking sequences of the SIR model in the six real networks, where the infection rate $\alpha =1.05\times \alpha _{th}$ in the SIR model.

Network	Degree	K-shell	WKSDN	CHB	MCDE	EHCC
Dolphins	0.8003	0.7409	0.8145	0.7730	0.7657	**0.9037**
Celegans	0.8030	0.7621	0.8170	0.7886	0.7664	**0.9060**
Email	0.7894	0.8131	0.8031	0.7856	0.8246	**0.9227**
Power	0.5979	0.5575	0.6570	0.7309	0.7485	**0.8325**
GrQc	0.6835	0.6967	0.7955	0.7590	**0.8253**	0.8205
PGP	0.6178	0.6545	0.7033	0.7425	0.7657	**0.7816**

The highest and second-highest values in each network are highlighted in bold and underlined, respectively.

#### Results of resolution

**Figure 2:**
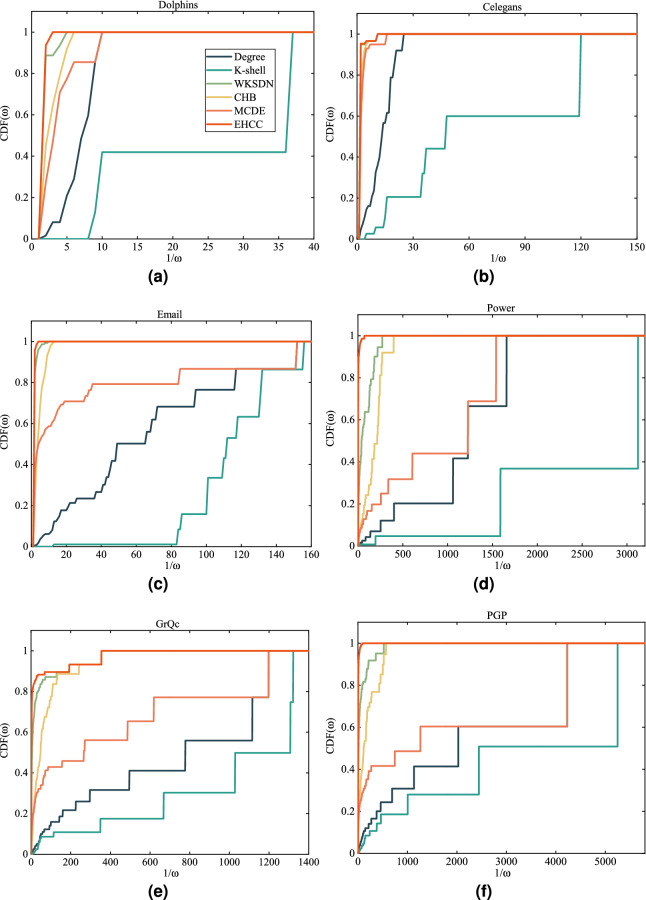
CDF (The cumulative distribution function) of different algorithms in six real networks. $\omega$ is the identity of the nodes. (**a**)–(**f**) are the networks Dolphins, Celegans, Email, Power, GrQc and PGP respectively.

Figure 2 presents the CDF curves (Eq. 7)of different algorithms in six real networks. It can be observed that the CCDF of EHCC increases the fastest, which illustrates that the node distribution given by EHCC is the best. In network Dolphins, EHCC performs best and WKSDN is second. In networks Celegans and Email, the CDF curves of EHCC and WKSDN almost overlap and are followed by that of MCDE. We also observe performance of EHCC is the best in network Power. Moreover, EHCC is far superior to other methods in networks Power and PGP. Table 7 shows the monotonicity (Eq. 8) of the different methods in the six real networks. The monotonicity scores of EHCC in all the networks except network GRQC were higher than 0.99. Obviously, the monotonicity of EHCC is the highest and K-shell is the worst, which supports the results in Fig. 2. In networks Dolphins, Email, GrQc, and PGP, the best performer is EHCC, followed by WKSDN. In the network Celegans, EHCC follows CHB, but still beats the other methods.

**Table 7:** Monotonicity scores of different algorithms in six real networks.

Network	Degree	K-shell	WKSDN	CHB	MCDE	EHCC
Dolphins	0.8312	0.3769	0.9905	0.9612	0.9243	**0.9979**
Celegans	0.9217	0.6094	0.9925	0.9939	0.9909	**0.9977**
Email	0.8874	0.8088	0.9994	0.9951	0.9460	**0.9999**
Power	0.5927	0.2459	0.9688	0.9273	0.6671	**0.9987**
GrQc	0.7459	0.6630	0.9841	0.9717	0.8417	**0.9872**
PGP	0.6193	0.4806	0.9872	0.9654	0.6753	**0.9992**

The highest and second highest values in each network are highlighted in bold and underlined, respectively.

## Discussion

This paper investigates the identification of important node in complex networks. In previous studies, degree centrality and K-shell decomposition were introduced as two benchmark methods for identifying important nodes. Degree centrality estimates the influence of a node based on its local characteristics (the number of neighbors). Whereas, K-shell evaluates the influence of a node according to its global characteristics (hierarchy position). However, a single local or global characteristic fails to effectively estimate the importance of the node. As a results, this paper proposes a multi-characteristic approach based on the extended degree and E-shell hierarchy decomposition. The extended degree improves the classical degree by introducing the neighbors’ degree. E-shell hierarchy decomposition is used to determine nodes’ position through the network’s hierarchical structure. Combining these two components (extended degree and position), we define a hybrid characteristic centrality (HCC) that can be a comprehensive indicator of the node’s importance. Furthermore, we propose an extended version of HCC called EHCC. As the numerical results listed in Tables 6, 7, and Fig. 2, the accuracy and resolution of the new approach are superior to those of the five well known approaches within the SIR model and the monotonicity function. In addition, it is observed that multi-characteristic methods (EHCC and MCDE) outperform single-characteristic methods (Degree and K-shell). The work in this paper provides new ideas for learning about and analyzing important nodes. However, some challenges still need to be further investigated. For example, the relationship between local and global features of nodes has not been well-documented. As a result, we will continue to explore new methods in future.

## Data Availability

All relevant data are available at https://networkrepository.com/.

## Code availability

The code covering this article can be accessed via Github.
